# Activation of adenosine A3 receptor attenuates progression of osteoarthritis through inhibiting the NLRP3/caspase‐1/GSDMD induced signalling

**DOI:** 10.1111/jcmm.17438

**Published:** 2022-06-30

**Authors:** Hui Bai, Zhiheng Zhang, Lin Liu, Xinyu Wang, Xiaopeng Song, Li Gao

**Affiliations:** ^1^ Heilongjiang Key Laboratory for Animal Disease Pathogenesis and Comparative Medicine, College of Veterinary Medicine Northeast Agricultural University Harbin China

**Keywords:** A3AR, NLRP3, osteoarthritis, pain, pyroptosis, ROS

## Abstract

The specific adenosine A3 receptor (A3AR) agonist (CF101) has potential for inflammation and pain in various disease, such as arthritis, cancer and neuropathic pain, while the role of A3AR in post‐traumatic OA and the underlying mechanism is largely unknown. CF101 was orally administrated in OA rats induced by anterior cruciate ligament transection (ACLT) surgery, and the rat primary chondrocytes were stimulated by hydrogen peroxide (H_2_O_2_, 300 μM). Histologic grading system was performed for detecting cartilage degeneration and immunohistochemistry for determining pyroptosis. The moleculars associated with cartilage homeostasis and inflammatory cytokines were analysed; moreover, the activation of NLRP3 inflammasome was determined. CF101 treatment significantly attenuated OA cartilage damage, OA‐related pain and cartilage pyroptosis. Chondrocytes stimulated by H_2_O_2_ evoked ROS release, thereby promoting the activation of NLRP3 inflammasome and facilitating the cleavage of GSDMD, which ultimately resulted in the mass release of pro‐inflammatory cytokines including IL‐1β and IL‐18, and production of matrix hydrolase. The pre‐treatment with CF101 powerfully inhibited the above process both in vivo and in vitro. Our findings demonstrated that activation of A3AR attenuates OA progression and relieves pain perception through suppression of cartilage degradation and inhibition of ROS/NLRP3/GSDMD signalling, indicating pyroptosis is a potential candidate for OA treatment.

## 1 INTRODUCTION

Osteoarthritis (OA) is a complex and multifactorial disease that is characterized by chronic joint pain and disability.^1^ Loss and abnormal remodelling of cartilage extracellular matrix (ECM), which is triggered by hydrolytic enzymes, are central to OA.^1^, ^2^ The specific mechanisms that lead to the development of OA are complicated, and several studies have demonstrated that chronic and low‐grade inflammation is associated with both OA pain and pathogenesis.^3^, ^4^ Unfortunately, there are still no effective disease modification drugs and therapies that can prevent OA progression. Therefore, the final strategy is total joint replacement.

In recent years, there have been numerous molecules and signalling pathways that have been found to be involved in cartilage disorders, which include pyroptosis cell death (a type of regulated cell death that is closely related to inflammation).^5^ Pyroptosis occurs depending on the cleavage of procaspase‐1 to yield active caspase‐1. Inflammasomes are multimeric protein complexes that assemble in the cytosol and act as platforms for caspase activation.^6^ Activation of nucleotide‐binding and oligomerization domain‐like receptor containing protein 3 (NLRP3) promotes the cleavage of caspase‐1 and further proteolytically matures proIL‐1β and proIL‐18 to their active forms, resulting in pyroptosis through gasdermin D (GSDMD) cleavage.^7^ IL‐1β, IL‐18 and TNF‐α are the main inflammatory mediators that are involved in the pathophysiology of OA, which lead to suppressing expression of type II collagen and aggrecan,^8^ and stimulates the release of matrix metalloproteinase (MMP)‐3 and MMP‐13.^9^ This indicates that NLRP3‐induced pyroptosis may contribute to OA progression by releasing pro‐inflammatory cytokines. Additionally, increasing evidence suggests that the NLRP3 inflammasome is involved OA pathogenesis, which leads to the synovial inflammation^10^ and cartilage degradation. In the synovial tissue of OA model rats, it was proved that the specific caspase1 inhibitor Ac‐YVAD‐CMK inhibited NLRP3 inflammasome‐induced pyroptosis and the expression of pro‐inflammatory factors IL‐1β, IL‐18, as well as alleviated OA pathology.^11^ Moreover, fibroblast‐like synoviocytes that were stimulated with LPS + ATP resulted in cell pyroptosis, while NLRP3 siRNAs attenuated this effect. These results suggested that LPS‐induced fibroblast‐like synoviocytes pyroptosis may be mediated by NLRP3 inflammsomes.^12^ In addition, Li et al. found that the P2X7 receptor antagonist (A740003) ameliorated monosodium iodoacetate‐induced cartilage degradation and OA‐like pyroptotic inflammation by rescuing the expression of P2X7, MMP13, NF‐κB p65, NLRP3, caspase‐1 and IL‐1β upregulation.^13^ In human, nucleus pulposus stimulated by lactate solution showed NLRP3 inflammasome activation and increased levels of pyroptosis, furthermore degeneration of the extracellular matrix. While lactate‐induced NLRP3 inflammasome activation was blocked by ASIC inhibitors and NLRP3 siRNA,^14^ these studies indicated the role of pyroptosis in OA cartilage.

The purine nucleoside adenosine is a naturally occurring multifunctional signalling molecule that has potential for managing inflammatory joint disease.^15^, ^16^ Adenosine mediates its effects through binding and activating one or more of four G protein‐coupled receptors expressed on the cell membrane: A1, A2A, A2B and A3.^17^ Currently, a large number of studies have focused on the role of adenosine A2A receptor and adenosine A3 receptor in articular cartilage. Evidence indicated that the loss of glycosaminoglycans, collagen and extracellular matrix occurred in A2A knockout mice, which was accompanied by increased expression of MMPs, cartilage cell hypertrophy and osteophyte formation.^18^ In addition, Liu Xiuling et al. (2019) found that the expression of IL‐6, MMP‐13 and type X collagen was significantly reduced after the use of adenosine‐coupled nanoparticles, which was partially reversed by A2A receptor antagonist (ZM 241385) in primary chondrocytes of mice stimulated with IL‐1β.^19^ These studies suggested that adenosine A2A receptor is directly involved in the maintenance of cartilage homeostasis. Furthermore, activation of adenosine A3A receptor (A3AR) has been documented to exhibit powerful anti‐inflammatory activity in a variety of diseases, including bowel inflammation,^20^ psoriasis^21^ and rheumatoid arthritis (RA).^22^ Numerous A3AR agonists and antagonists have been tested for disease intervention. It has been reported that oral administration of CF101 (a selective A3AR agonists) downregulated levels of TNF‐a, and increased apoptosis in infiltrating macrophages.^23^ A3AR ablation in aged mice exhibited abnormal cartilage metabolism and early signs of OA, which include damage upon the superficial layer of cartilage matrix with upregulated expressions of MMP13 and a disintegrin and metalloprotease with thrombospond in type 5 motifs (ADAMTS‐5).^24^ Given the potential role of A3AR as an anti‐inflammatory target, we thus tested the effect of CF101 on OA in a post‐traumatic rat model. Our results demonstrated that the activation of A3AR by CF101 can attenuate severity of OA induced by ACLT in rats by suppressing the NLRP3‐induced pyroptosis and downstream inflammatory cascades.

## 2 MATERIALS AND METHODS

### 2.1 Reagents

CF101, MCC950 and VX765 were purchased from MedChemExpress. MRS1523 was purchased from ApexBio. A stock solution was prepared in DMSO and was further diluted in 0.9% (wt/vol) sterile saline solution or in cell culture medium.

### 2.2 Animals

Sprague‐Dawley (SD) rats (bought from Liaoning Changsheng Biotechnology Co., Ltd.) were raised at the experimental animal centre on a standard 12 h dark/light cycle. All experimental procedures and protocols were carried out in accordance with the guidelines for Experimental Animal Ethics Committee of Northeast Agricultural University.

### 2.3 Rat model of surgically induced OA

Anterior cruciate ligament transection (ACLT) model was induced using 10‐week‐old male rats. Rats accepted ACLT surgery y (*n* = 48) were randomly divided into 4 groups (12 rats per group), (1) ACLT group (vehicle), (2) ACLT+CF101 treatment group (100 μg/kg given twice daily), (3) ACLT+MRS1523 treatment group (100 μg/kg given twice daily) and (4) ACLT+MRS1523 + CF101 group (MRS1523 was administrated 30 min before CF101 treatment). Sham surgery was performed on the right knee joints from a separated group of rats as control (*n* = 12, treatment with vehicle). All treatment was started on Week 1 after ACLT surgery by gavage and was continued until the study was terminated on the last day of Week 6. The dose of each drug we used was based on previous study.^23^

### 2.4 Pain assessment

Secondary mechanical allodynia was assessed with Von Frey filaments (Aesthesio) in a blinded fashion. Mechanical allodynia was measured using the simplified up‐down method^25^ (*n* = 12 per group) in which Von Frey filaments were pressed against the plantar surface of the paw until the filaments flexed and held for 6–7 s for estimating paw withdrawal threshold (PWT). Filaments numbered 7 through 14 (0.60–10 g) were used and testing always began with filament 10. If there was a positive response, the next lower strength hair was applied; if no withdrawal was present, the next higher value was applied. A total of 5 stimulus presentations occurred. Each animal went through two test rounds and whole numbers was used to represent the filament value.

After sacrifice, serum and urine samples were collected from 5 groups. After that, six rats in each group were performed for histological section, and another six rats in each group were performed for collecting synovial fluid.

### 2.5 Histology and Immunohistochemistry

Knee joint specimens were extracted from the rats (*n* = 6 per group), fixed in 4% paraformaldehyde and decalcified in decalcification solution (Servicebio Biotechnology Co., Ltd, G1105). The joints were then embedded in paraffin and cut in 4 μm thick section. Slides were stained with haematoxylin and eosin (HE) and Safranin O/Fast Green. Histological scores of sections were performed by two experienced observers. The degree of cartilage degeneration was graded according to the Osteoarthritis Research Society International (OARSI) grading system.^26^

For immunohistochemistry (IHC) experiment (*n* = 3 per group), the sections were incubated with (1) anti‐rabbit polyclonal antibody directed at Collagen II (1:100; Abcam, ab34712), or (2) anti‐ MMP‐13 mouse monoclonal antibody (1:150; Novus, OTI2D8), or (3) anti‐ ADAMTS5 rabbit polyclonal antibody (1:100; Gene Tex, GTX123657), or (4) anti‐ Aggrecan rabbit polyclonal antibody (1:100; Absin, abs121273), or (5) anti‐ NLRP3 rabbit polyclonal antibody (1:200; ABclonal, A12694), or (6) anti‐ caspase‐1 rabbit polyclonal antibody ((1:150; ABclonal, A0964), or (7) anti‐ASC rabbit polyclonal antibody (1:100, Wanlei, WL02462), or anti‐GSDMD rabbit polyclonal antibody (1:100, Affinity, AF4012), or (8) anti‐adenosine A3 receptor rabbit polyclonal antibody (1:200, Absin, abs121848). Horseradish peroxidase (HRP)‐conjugated secondary antibody was applied and stained with diaminobenzidine (DAB) kit. The positive stained chondrocytes in three central regions of articular cartilage were counted in a blinded fashion. Integrated optimal density (IOD) was semi‐quantified using Image‐Pro Plus software, v.6.0.

### 2.6 ELISA

Serum samples, synovial fluid lavage fluids and urine samples were collected (*n* = 6 per group) and concentrations of IL‐1β, IL‐18, COX‐2, CTX‐II, COMP and GAG were measured using specific rat enzyme‐linked immunosorbent assay (ELISA) kits (Jingmei, Co., Ltd.) according to the protocol provided by the manufacturer. All of the samples were assessed in triplicate.

### 2.7 Rat primary chondrocytes culture and stimulation

Rat cartilage from femoral condyle, tibial plateau and caput femoris were obtained from lactating rats (14‐21‐day‐old). After trypsinization (0.25%, GIBCO, USA) of cartilage for 30 min, the tissues were cut into small pieces and then digested at 37°C with 0.2% collagenase II (BioFroxx, Germany) for 4 h. The primary chondrocytes were cultured with DMEM/F12, including 10% foetal calf serum (Biological Industries).

For induction of NLRP3 inflammasome activation, chondrocytes were cultured until 60%–70% confluence, and pre‐incubated with CF101 (1 μM) for 24 h, or pre‐incubated with MRS1523 (2 μM) for 2 h before treatment with CF101. After that, H_2_O_2_ (300 μM) was added into culture medium for another 24 h.

MCC950 (10 μM) and VX765 (50 μM), inhibitors of NLRP3 and caspase‐1, respectively, were also pre‐treated for 1 h, before treatment with H_2_O_2_ for 24 h.

### 2.8 LDH assay

Chondrocytes were plated in 96‐well plates before exposure to different drugs (the same as 2.7). The levels of LDH in cell culture supernatants released from injured chondrocytes were determined using LDH Release Assay Kit (Beyotime) following the manufacturer's instruction (*n* = 6). The absorbance was measured at a wavelength of 490 nm.

### 2.9 Real‐time quantitative polymerase chain reaction (RT‐qPCR)

Total RNA was extracted using the RNAsimple Total RNA Kit (TianGen Biotechnology, Co., Ltd). Synthesis of cDNA was obtained using GoScript™ Reverse Transcription Mix, Oligo (dT) (Promega,). Real‐time quantitative PCR was performed in 10 μl reaction system containing 5 μl SYBR SuperMix (Thermo Fisher Scientific), 4.6 μl diluted cDNA, 0.2 μl forward primer and 0.2 μl reverse primer, and to determine the relative gene expression of NLRP3, ASC, caspase‐1 and GSDMD, with an endogenous control of glyceraldehyde‐3phosphate dehydrogenase (GAPDH). Primer sequences (Sangon Biotech, Shanghai, Co., Ltd.) are shown in Table 1. The expression of genes was analysed by method of 2^−ΔΔCt.^ (*n* = 3).

**TABLE 1:** Primer sequences for genes tested with primary chondrocytes of rat

Gene	Forward	Reverse
GAPDH	5′‐GATGCCCCCATGTTTGTGAT‐3′	5′‐GGCATGGACTGTGGTCATGAG‐3′
ASC	5′‐GATGCCATCCTGGACGCTCTTG‐3′	5′‐ATGAGTGCTTGCCTGTGTTGGTC‐3′
Caspase‐1	5′‐GCCGTGGAGAGAAACAAGGAGTG‐3′	5′‐GGTCACCCTTTCAGTGGTTGGC‐3′
GSDMD	5′‐GTCTGCTTGCCGTACTCCATTCC‐3′	5′‐AGGCTCTACCTGCTCACCACTG‐3′
NLRP3	5′‐CTGCGGACTGACCCATCAATGC‐3′	5′‐ACCAATGCGAGATCCTGACAACAC‐3′

### 2.10 Western blot

Total protein from chondrocytes were extracted, and protein concentration was determined using BCA Protein Assay Kit (Beyotime; Shang Hai, China). Equal amount of protein samples (30 μg) was separated by 10% or 12% sodium dodecyl sulphate polyacrylamide gel electrophoresis (SDS‐PAGE) and transferred to a BioTrace NT Nitrocellulose transfer membrane (Pall). The membranes were than blocked with 5% non‐fat milk in tris buffered saline tween buffer for 1 or 2 h, and then incubated with primary antibodies against NLRP3 (1:1000, ABclonal, A12694), ASC (1:1000, ABclonal, A16672), caspase‐1 (1:1000, ABclonal, A0964), GSDMD (1:2000, ABclonal, A10164), MMP‐13 (1:1000, Novus, OTI2D8), ADAMTS‐5 (1:1000, Gene Tex, GTX123657), Adenosine A3 receptor (A3AR, 1:1000, Absin, abs121848) and GAPDH (1: 1000, ABclonal, A19056) overnight at 4°C. After that, rabbit HRP‐conjugated secondary antibody (1:3000, ZSGB‐BIO, ZB2306) was added and incubated at room temperature for 2 h. Intensity values were analysed using the ImageJ software (*n* = 3).

### 2.11 Flow cytometry analysis of caspase‐1 activity

Pyroptosis in chondrocytes was measured by FAM‐FLICA caspase‐1 (YVAD) assay kit (ImmunoChemistry). FAM‐YVAD‐FMK enters each cell and irreversibly binds to activated caspase‐1 and determined by flow cytometry. Briefly, 1.0 × 10^6^ cell suspension was incubated with FAM‐YVAD‐FMK for 60 min at 37°C in dark conditions. After centrifugation, the supernatant was removed and the cell pellet was washed twice with 1 × wash buffer. Cells were resuspended in PI staining buffer. The cells were analysed using an BD FACSAria flow cytometerr (Becton, Dickinson and Company), and pyroptosis was defined as double positive for FAMYVAD‐ FMK and PI staining (*n* = 3).

### 2.12 Measurement of ROS production

Production of intracellular ROS was detected by the fluorescent probe DCFH‐DA using ROS Assay Kit (Beyotime). After treatment, chondrocytes were incubated with 10 μM DCFH‐DA at 37°C for 20 min in the dark. The culture medium was washed three times with PBS, harvested by trypsinization, and the fluorescence intensity was detected using a Spark Multi‐mode microplate readers (Tecan, excitation at 488 nm, emission at 525 nm) and images were performed with microscopy fluorescence (*n* = 6).

### 2.13 Intracellular potassium or calcium detection

After treatment, the cells were collected and broke through repeated freezing and thawing methods, then centrifuge and collect the supernatant. Intracellular potassium was detected by Potassium Assay Kit (Nanjing Jiancheng Bioengineering Institute), following the manufacturer's instructions (*n* = 6).

After treatment, cells were loaded with Ca^2+^ indicator dye by incubating the culture dish in standard solution supplemented with 5 μM Fluo‐3‐AM (Beyotime Biotechnology) for 30 min at 37°C; then, the incubation was washed by PBS for two times. Absorbance was tested using Tecan Multi‐Mode Microplate Readers (*n* = 6).

### 2.14 Statistical analysis

All data were performed using SPSS 22.0 software or GraphPad Prism 8.3.0 software, and results are expressed as the mean ± standard error of mean (SEM). Statistical analyses were one‐way analysis of variance (anova) was performed followed by Tukey's honestly significant difference post hoc test to compensate for multiple pairwise comparisons, and *p* < 0.05 was considered statistically significant.

## 3 RESULTS

### 3.1 Activation of A3AR inhibits ACLT‐induced pain and reduces COX‐2 expression

**FIGURE 1:**
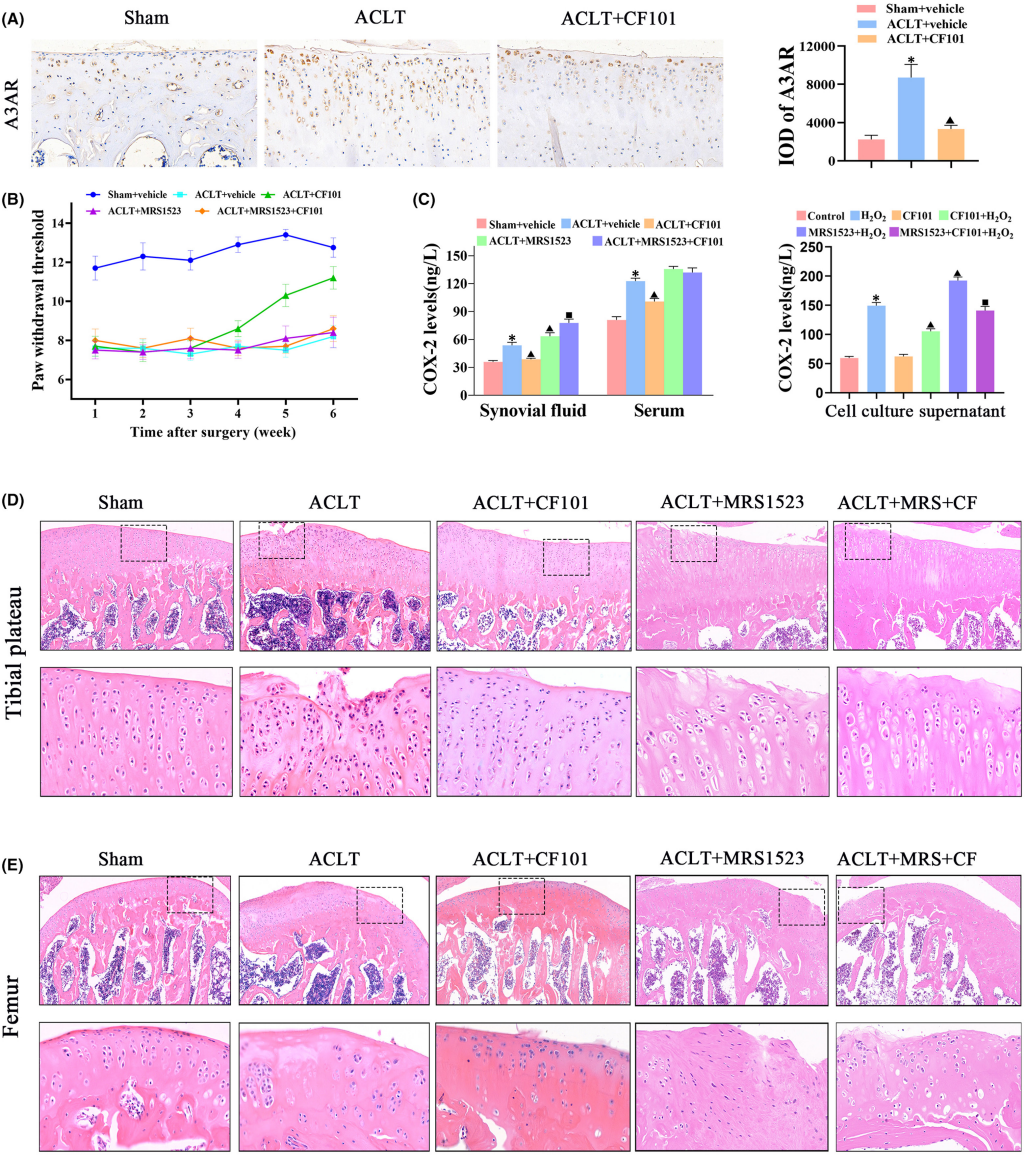
A3AR activation by CF101 ameliorated OA‐related pain and protected the structure of cartilage. (A) IHC analysis of A3AR in tibial plateau of right knee joints (*n* = 3, the positive stained chondrocytes in three central regions of articular cartilage were counted using Image Pro Plus version 6.0 software), Original magnification × 200. (B) Effect of CF101 on von Frey hair withdrawal threshold in ACLT‐induced rats (*n* = 12). (C) ELISA assay of COX‐2 in synovial fluid, serum and cell culture supernatant (*n* = 6). (D) The pictures above are representative images of cartilage in tibial plateau (right knees of rats) stained by hemotoxylin and eosin (HE), Original magnification × 100. The picture below is expansion of the region occupied by articular cartilage (*n* = 6), Original magnification × 400. (E) The pictures above are representative images of cartilage in femur (right knees of rats) stained by hemotoxylin and eosin (HE), Original magnification × 100. The picture below is expansion of the region occupied by articular cartilage (*n* = 6), Original magnification × 400. Values are the mean ± SEM; *p < 0.05 vs. Sham group. ^▲^ *p* < 0.05, vs. ACLT group. ^■^ *p* < 0.05, vs. ACLT + CF101 group

In order to determine the role of A3AR in post‐traumatic OA, the knee joints were collected from rats that underwent ACLT surgery. The expression of A3AR increased in cartilage after ACLT induction, while CF101 treatment downregulated the expression of A3AR (Figure 1A). Moreover, in the group that received ACL transection, the PWT values showed a clear reduction when compared with the vehicle group at one‐week post‐surgery, and the pain lasted until 6 weeks after surgery. Oral administration of CF101 had no obvious effect on PWT at 1–3 weeks after surgery. Effects of CF101 on ACLT‐induced lowering of PWT were found to be less immediate and were significant on week 4 post‐surgery. Considering the specificity of the A3AR in relieving OA pain, MRS1523, a selectively antagonist of A3AR, was provided prior to CF101 treatment. The effect of CF101 on PWT was reversed via MRS1523, and the PWT was decreased at 4–6 weeks (Figure 1B). The ACLT‐induced model also showed increased serum and synovial fluids levels of COX‐2, which is a biomarker of OA pain, compared to the sham control group. The rat primary chondrocytes stimulated by H_2_O_2_ exhibited elevated COX‐2 levels in cell culture supernatant. Administration of CF101 significantly decreased levels of COX‐2, while the effect was reversed by MRS1523 both in vivo and in vitro (Figure 1C).

### 3.2 Activation of A3AR ameliorates ACLT‐induced OA progression in rats

**FIGURE 2:**
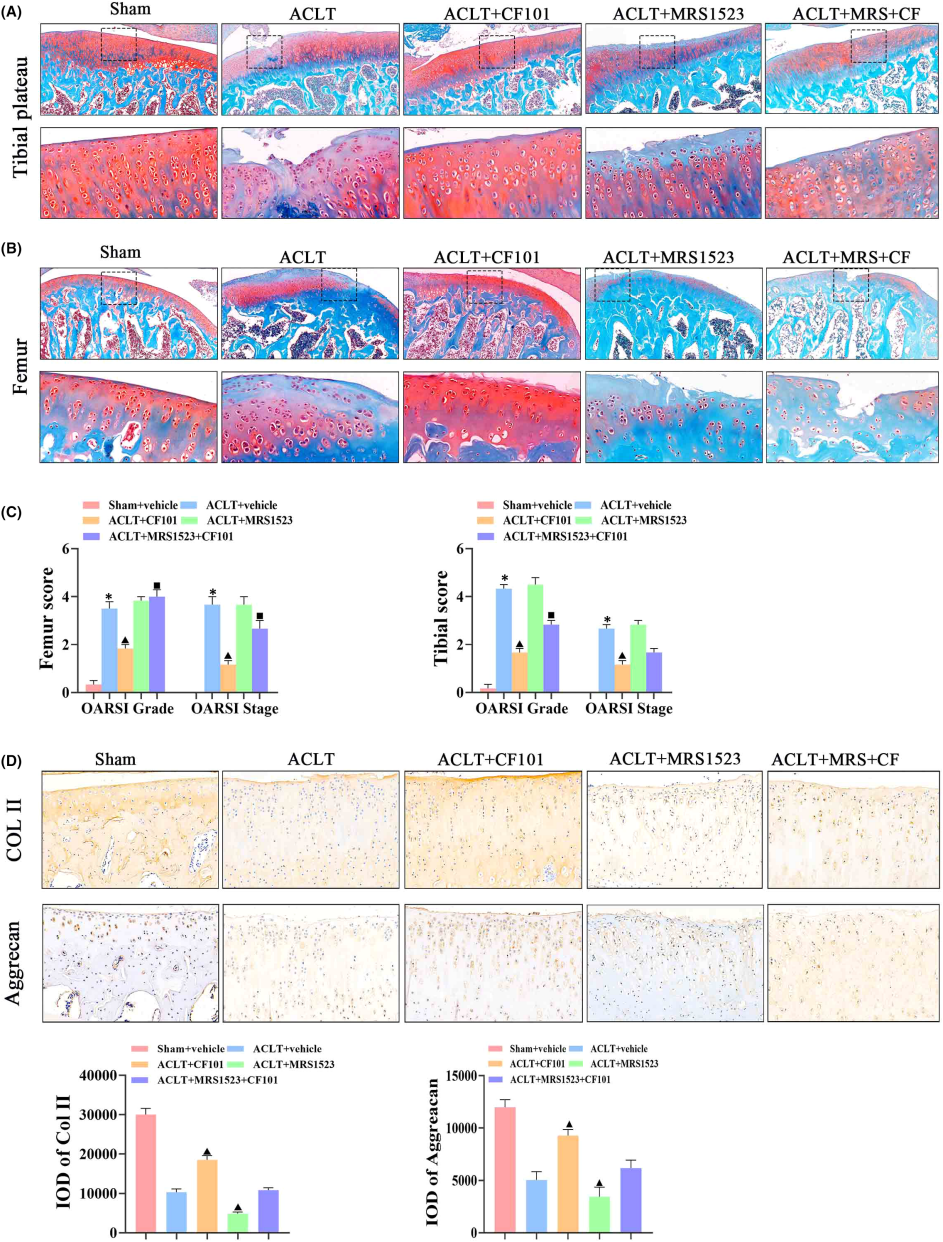
CF101 treatment prevented cartilage degeneration through promoting production of main components of cartilage extracellular matrix. (A) Safranin O‐fast green staining was performed to evaluated the relative content of proteoglycan and articular cartilage degeneration. The picture above is representative images of cartilage in tibial plateau (right knees of rats) stained by Safranin O‐fast green, Original magnification × 100. The picture below is expansion of the region occupied by articular cartilage (*n* = 6), Original magnification × 400. (B) The pictures above are representative images of cartilage in femur (right knees of rats) stained by Safranin O‐fast green, Original magnification × 100. The picture below is expansion of the region occupied by articular cartilage (*n* = 6), Original magnification × 400. (C) Scores of tibial and femur assessed by OARSI Scoring System, including OA grade (0–6) and OA stage (0–4) (*n* = 6). (D) IHC analysis of Col II and Aggrecan in tibial plateau (*n* = 3, the positive stained chondrocytes in three central regions of articular cartilage were counted using Image Pro Plus version 6.0 software), Original magnification × 200. Values are the mean ± SEM; *p < 0.05 vs. Sham group. ^▲^ *p* < 0.05, vs. ACLT group. ^■^ *p* < 0.05, vs. ACLT + CF101 group

Next, we investigated the effect of CF101 on cartilage degeneration in experimental OA rats. CF101 administration protected the structure of cartilage in OA through HE staining (Figure 1D,E). Safranine O staining showed that ACLT surgery induced an obvious surface discontinuity, matrix disturbance, disordered chondrocytes, hypertrophic chondrocytes, vertical cracks and increased OARSI score (Figure 2A–C). Additionally, the expression of aggrecan and col II was downregulated in ACLT group (Figure 2D). Compared to the ACLT rats, the OARSI score was significantly decreased, while the expression of aggrecan and col II was upregulated in the CF101 administration group. All of these changes were reversed by MRS1523 pre‐treatment, suggesting the ACLT‐induced cartilage degeneration and mechanical hyperalgesia were mediated by A3AR.

### 3.3 Activation of A3AR reduces extracellular degradation in ACLT‐induced OA and in H_2_O_2_ ‐treated chondrocytes

**FIGURE 3:**
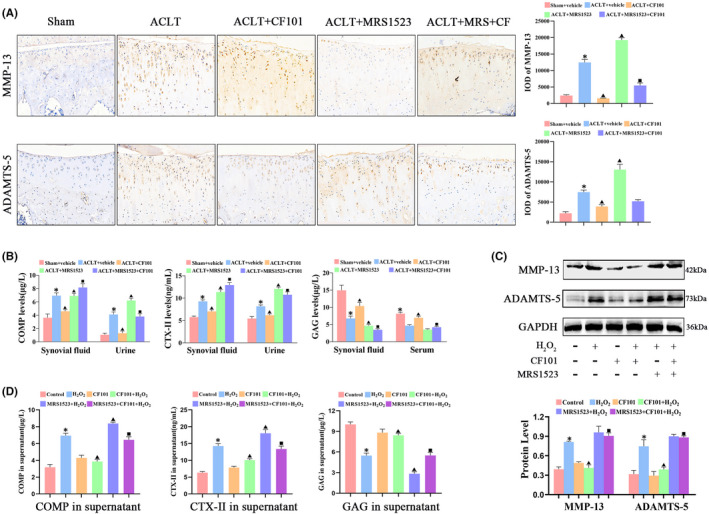
CF101 treatment inhibited catabolism of articular cartilage in ACLT‐induced rats and H_2_O_2_‐stimulated chondrocytes. (A) IHC analysis of MMP‐13 and ADAMTS‐5 in tibial plateau of cartilage in vivo (*n* = 3, the positive stained chondrocytes in three central regions of articular cartilage were counted using Image Pro Plus version 6.0 software), Original magnification × 200. (B) Elisa assays of COMP, CTX‐II and GAG in synovial fluid, serum and urine in vivo (n = 6). (C) Protein levels of MMP‐13 and ADAMTS‐5 in chondrocytes induced by H_2_O_2_ in vitro through Western blot (*n* = 3). (D) Elisa assays of COMP, CTX‐ II and GAG in cell culture supernatant induced by H_2_O_2_ in vitro (*n* = 6). Values are the mean ± SEM; *p < 0.05 vs. Sham group/Control. ^▲^ *p* < 0.05, vs. ACLT group/ H_2_O_2_ group. ^■^ *p* < 0.05, vs. ACLT + CF101 group/CF101 + H_2_O_2_ group

To evaluated the role of A3AR in extracellular degradation induced by ACLT, we determined the expression of MMP‐13 and ADAMTS‐5. The expression of MMP‐13 and ADAMTS‐5 in the cartilage was increased in the ACLT group when compared with sham group, while CF101 treatment decreased the expression of MMP‐13 and ADAMTS‐5 (Figure 3A). Moreover, COMP and CTX‐ II, which are potential biomarker for OA diagnosis, were also decreased in the CF101‐treated group, both in urine and synovial fluids, compared to the ACLT group. However, the low levels of GAG showed contrary tendency. The changeover occurred in the MRS1523 pre‐treated rats (Figure 3B).

In order to mimic OA conditions, H_2_O_2_ were utilized to stimulate chondrocytes in the in vitro study. H_2_O_2_‐induced chondrocytes demonstrated upregulated protein expression of MMP‐13 and ADAMTS‐5 in chondrocytes, as well as increased levels of COMP and CTX‐ II, instead of decreased expression of GAG in the cell culture supernatant. Similarly, the effect of CF101 and MRS1523 was consistent with the in vivo experiment (Figure 3C,D).

### 3.4 Activation of A3AR ameliorate cartilage damage through inhibiting NLRP3‐induced pyroptotic cell death

To determine the role of NLRP3/caspase‐1 induced pyroptosis in chondrocytes homeostasis, we used MCC950 (a selective inhibitor of NLRP3) and VX765 (a selective inhibitor of caspase‐1), respectively, in a H_2_O_2_‐induced in vitro model. The results showed that both MCC950 and VX765 inhibited H_2_O_2_ evoked protein and gene expression of NLRP3, caspase‐1 and GSDMD (Figure S1a–d), LDH release (Figure S1e), and the expression levels of IL‐1β and IL‐18 in the cell culture supernatant (Figure S1f,g). Moreover, MCC950 and VX765 decreased the expression of COMP and CTX‐ II, while increased the levels of GAG in the cell culture supernatant (Figure S1h–j).

**FIGURE 4:**
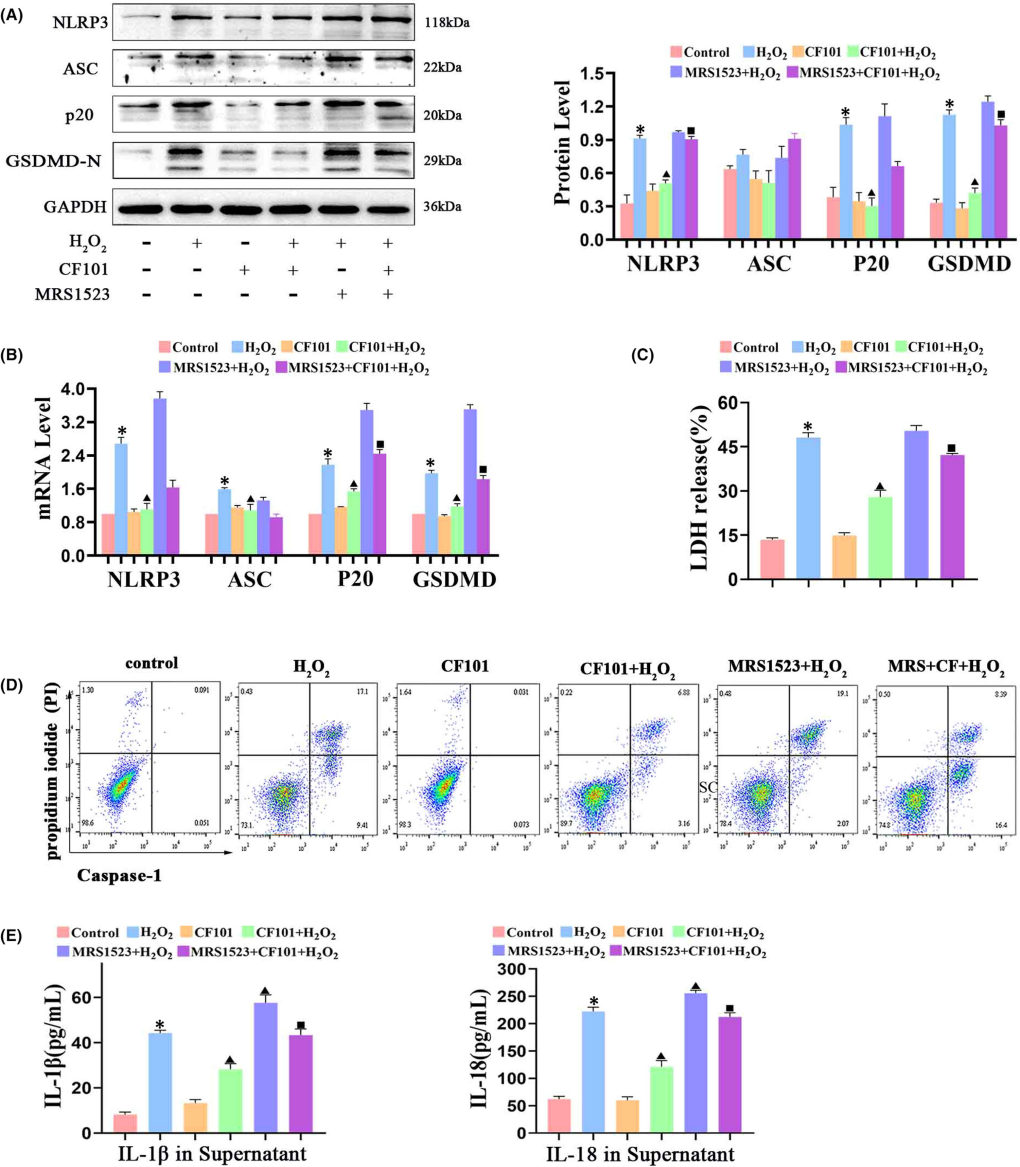
Activation of A3AR (CF101 pre‐treatment) inhibited NLRP3/caspase‐1/GSDMD induced pyroptosis in chondrocytes in vitro. (A) Protein levels of NLRP3, ASC, cleaved caspase‐1 (p20) and GSDMD‐N in H_2_O_2_‐stimulated chondrocytes through Western blot (*n* = 3). (B) Real‐time PCR analysis of gene expression of NLRP3, ASC and caspase‐1 and GSDMD in H_2_O_2_‐stimulated chondrocytes (*n* = 3). (C) Cell death was measured by LDH release assay kit (*n* = 6). (D) Flow cytometry analysis was used to measure caspase‐1 activity in chondrocytes. (E) Elisa assay of IL‐1β and IL‐18 in cell culture supernatant (*n* = 6). Values are the mean ± SEM; *p < 0.05 vs. Control group. ^▲^ *p* < 0.05, vs. H_2_O_2_ group. ^■^ *p* < 0.05, vs. CF101+ H_2_O_2_ group

**FIGURE 5:**
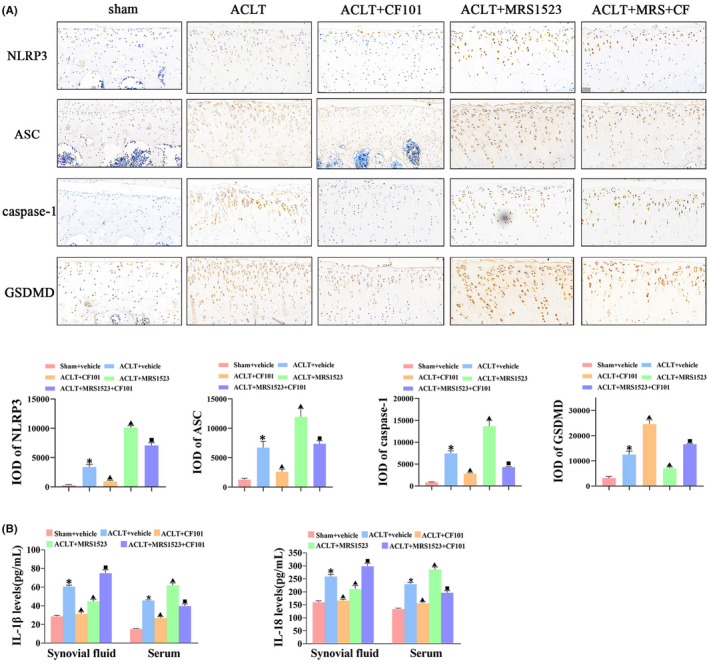
Activation of A3AR inhibited GSDMD cleavage and production of pro‐inflammatory cytokines in articular cartilage in vivo. (A) IHC analysis of NLRP3, ASC, caspase‐1 and GSDMD in tibial plateau of cartilage (*n* = 3, the positive stained chondrocytes in three central regions of articular cartilage were counted using Image Pro Plus version 6.0 software), Original magnification × 200. (B) Elisa assay of IL‐1β and IL‐18 in synovial fluid and serum after CF101 treatment (*n* = 6). Values are the mean ± SEM; *p < 0.05 vs. Sham group. ^▲^ *p* < 0.05, vs. ACLT group. ^■^ *p* < 0.05, vs. ACLT + CF101 group

These results demonstrated that NLRP3‐induced pyroptosis participated in ECM degradation in chondrocytes, and activation of A3AR by CF101 treatment inhibited ECM degradation in articular cartilage. Therefore, we explored whether inhibition of pyroptosis was the central pathway that was involved in OA protection after activation of A3AR. The results showed that CF101 pre‐incubation remarkably decreased the protein and mRNA expression of NLRP3, ASC, caspase‐1 (p20) and GSDMD‐N, and reduced the LDH release in vitro (Figure 4A–C). Furthermore, the flow cytometry results of caspase‐1/PI staining showed that CF101 significantly reduced active caspase‐1 and inhibited H_2_O_2_‐induced pyroptosis in chondrocytes (Figure 4D). In addition, the expression of IL‐1β and IL‐18 was also decreased in the CF101‐treated group when compared with H_2_O_2_ group (Figure 4E). Consistently, the in vivo study showed that the immunohistochemistry positive staining of NLRP3, ASC, caspase‐1 and GSDMD were decreased after CF101 treatment when compared with ACLT group (Figure 5A). Moreover, CF101 treatment declined the levels of IL‐1β and IL‐18 in serum and synovial fluids (Figure 5B). Notably, administration of MRS1523 counteracted the effects of CF101 both in vitro and in vivo.

### 3.5 CF101 suppresses NLRP3 inflammasome activation through inhibiting the production ROS in chondrocytes

**FIGURE 6:**
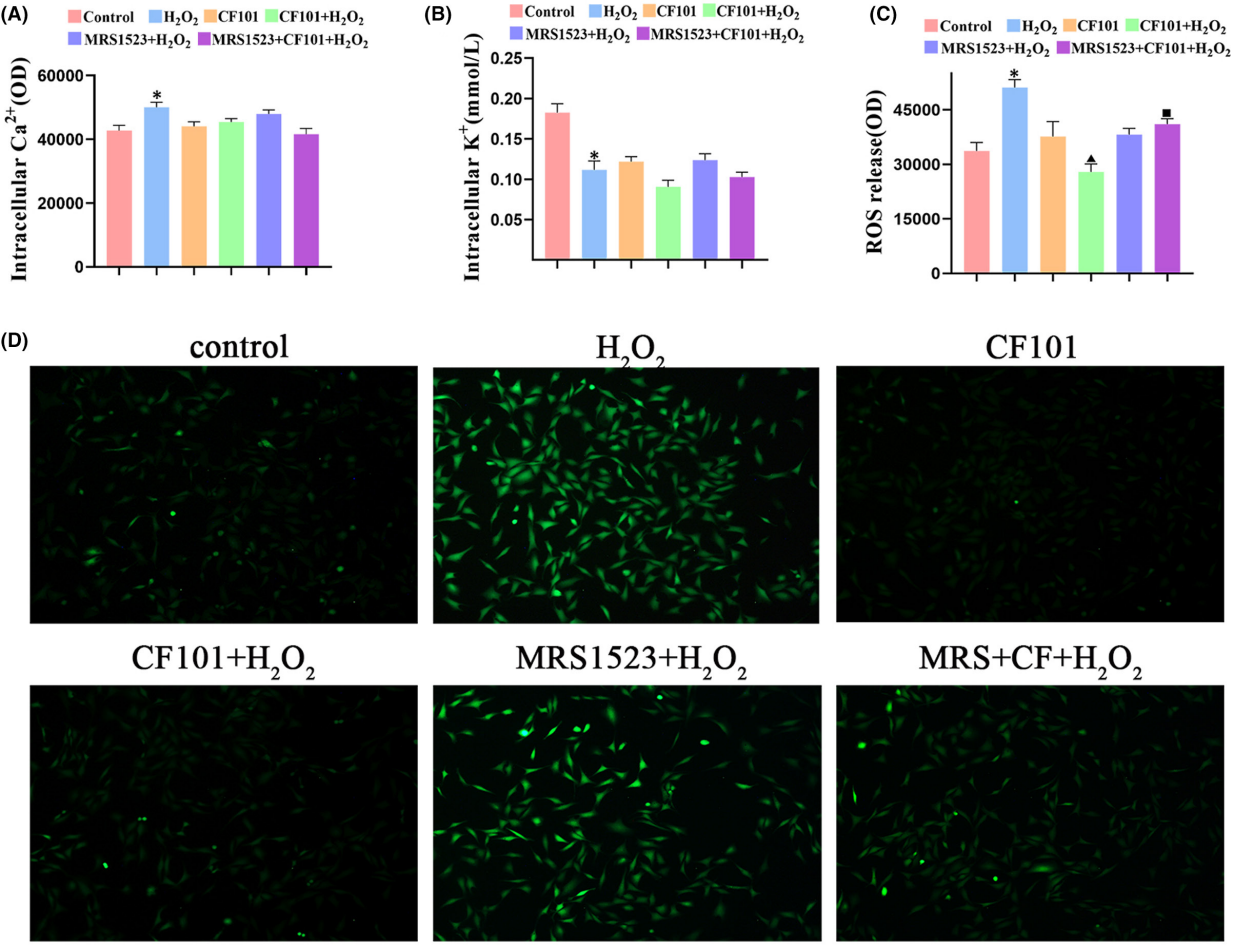
CF101 treatment inhibited NLRP3 activation through reducing ROS release. (A–C) Fluorescence intensity of the Ca^2+^, K^+^ and ROS were measured in chondrocytes through microplate readers (*n* = 6). (D) Fluorescence probe was used to label ROS, and the image was obtained by fluorescence microscope, Original magnification × 100. Values are the mean ± SEM; *p < 0.05 vs. Control group. ^▲^ *p* < 0.05, vs. H_2_O_2_ group. ^■^ *p* < 0.05, vs. CF101+ H_2_O_2_ group

Several events have been proposed to clarify the activation of the NLRP3 inflammasome. ROS, potassium (K^+^) efflux and calcium (Ca^+^) afflux were reported to contribute to the activation of NLRP3 inflammasome. We found that H_2_O_2_ stimulation led to an increase of ROS and Ca^2+^, but decreasing K^+^ in chondrocytes. CF101 treatment did not have an effect on the intracellular levels of K^+^ and Ca^2+^ (Figure 6A,B), while it did significantly suppress on the levels of ROS (Figure 6C,D). The results indicated that A3AR activation downregulated the NLRP3 inflammasome and pro‐inflammatory cascades by inhibition of production of ROS.

## 4 DISCUSSION

Currently, there are no curative therapeutics available for OA, and the underlying mechanism needs to be further explored for personalized treatment.^27^ Data from several groups agree on the therapeutic potential of activation of A2AR and A3AR in OA. Previous studies have found that osteoclast differentiation and chondrocyte inflammation were inhibited after activation of A2AR.^28^ In addition, aged A2A‐KO mice spontaneously developed OA, and exhibit abnormal chondrocyte hypertrophy, together with excessive marginal bone growth.^29^ Intra‐articular injections of adenosine prevented the development of OA in rats while the effect was abrogated by the co‐injection of ZM 241385 (a selective A2A receptor antagonist).^29^ These studies suggested that A2AR activation might be available to OA prevention. Additionally, activation of A3AR through CF101 treatment is believed to protect OA induced by monosodium iodoacetate (MIA) through inhibiting the NF‐κB signalling pathway in previous studies.^23^ Varani et al. verified that activation of A3AR (CF102) inhibited p38 MAPK and NF‐κB pathways, and the effect was abolished by selective adenosine antagonists.^30^ Fishman et al. also determined that the PI3K–NF‐κB pathway is involved in mediating the anti‐inflammatory effect of CF101 in adjuvant‐induced arthritis.^31^ Both adenosine A2A and A3 agonists have been extensively studied in pain management.^32^, ^33^ It is worth noting that A2A agonists were effective for chronic pain, however, accompanied with cardiovascular side effects.^32^, ^33^ A3AR agonists (CF101) is also effective in models of neuropathic pain induced by diverse chemotherapeutic agents,^33^ and demonstrated a good safety profile.^34^ Therefore, our study tested the effect of A3AR agonists on OA cartilage injury and pain, but not A2AR. The results presented here demonstrated that activation of A3AR by CF101 ameliorated the structural damage of articular cartilage and prevented ECM degradation in an ACLT‐induced OA rat model. We provide evidence that activation of A3AR exerts an OA prevention effect in post‐traumatic model in vivo. Furthermore, we observed that expression of A3AR increased in ACLT‐induced cartilage, whereas this expression was downregulated upon CF101 treatment. This was consistent with previous results, mainly due to the cellular response to the drug after using selective agonist treatment.^23^, ^35^

The development and progression of OA are now believed to involve inflammation, particularly in the early stages of the disease.^36^ Numerous studies implicated that the role of A3AR in regulating immune responses and inflammation. For example, A3AR agonists (CF102) decreased the production of TNF‐α and IL‐8, and inhibited p38 MAPK and NF‐kB pathways in human synoviocytes.^37^ Furthermore, CF101 was reported to ameliorate cartilage and bone destruction and decrease the expression of PI3K, PKB/Akt, IKK, NF‐κB, TNF‐α and RANK in experimental animal models of collagen and adjuvant‐induced arthritis (AIA).^38^ Selective A3 receptor activation is also known to protect human chondrocytes from cell apoptosis caused by pro‐inflammatory cytokines or hypo‐osmotic stress.^39^ Our present study confirms that activation of A3AR by CF101 inhibits inflammation in OA disease model, and the effects are associated with NLRP3‐induced pyroptosis.

NLRP3 is the best‐characterized inflammasome which induces an inflammatory, pyroptotic cell death.^40^ The role of NLRP3 in OA is controversial, mainly due to the production of NLRP3 by cartilage or synovial membrane. Several studies have reported that IL‐1β is involved in OA cartilage degradation, which may not be produced by chondrocytes but rather by synovial cells. Therefore, the synovial tissue might have effect on cartilage degradation via NLRP3 activation.^41^ Various studies have reported that pro‐inflammatory cytokines (i.e. IL‐1β, TNF‐α and IL‐6) involved in OA pathogenesis are released by chondrocytes, macrophages, and fibroblasts. These cytokines induced catabolic events such as promoting the production of MMPs, ADAMTS and pro‐inflammatory mediators (NO, PGE‐2).^42^, ^43^ However, IL‐1β or TNF‐α inhibition did not produce the desired effects of preventing OA progression.^44^, ^45^ Therefore, future studies should consider that the development of OA does not depend on a single cytokine; rather, the same signalling pathway can be activated by different cytokines, and the interaction of multiple factors plays a key role in the occurrence and development of diseases. As a major source of cytokine production, protein expression of NLRP3 was shown to be increased in OA patients compared to controls in samples taken from the synovial membrane.^46^ However, Cheng Fang et al^47^ and Yang Yue et al^48^ observed that there was an activation of NLRP3 inflammasome in articular cartilage tissue of OA rats. Recently, Ni Bowei and his colleagues found that MCC950 (a specific NLRP3 inhibitor) attenuates cartilage degeneration through inhibiting cartilage catabolism and regulating Nrf2/HO‐1/NQO1, PI3k/Akt/mTOR, P38/MAPK and JNK/MAPK pathways in OA, which demonstrated that NLRP3 is involved in cartilage disorders. Consistently, our presented data also confirm the protection effect of MCC950 in in vitro rat chondrocytes through regulating ECM degradation biomarkers, including COMP, CTX‐II and GAG. Furthermore, we also observed the protective effect of VX765 (a specific caspase‐1 inhibitor) in chondrocytes stimulated by H_2_O_2_, suggesting the NLRP3/caspase‐1 axis indeed participated in OA process.

Several events have been proposed to explain the activation of the NLRP3 inflammasome including the production of ROS, mitochondrial damage, Ca^2+^ signalling and cytosolic K^+^ efflux.^49^ In order to explore the key mechanism or signal of CF101 inhibits NLRP3‐induced pathway, we found that CF101 suppressed NLRP3 inflammasome activation via decreasing the release of ROS, without affecting Ca^2+^ and K^+^ levels in in vitro chondrocytes. During the OA process, excess levels of these ROS have been generally recognized to play key roles in the degeneration of articular cartilage function.^50^ Evidence indicated that oral administration of N‐acetyl cysteine (NAC) prevented osteoarthritis development and progression in an animal model.^51^ Furthermore, ROS‐induced oxidative stress was believed to be the main cause of NLRP3 inflammasome activation during OA development, and activating the Nrf2/HO‐1 signalling alleviates osteoarthritis development by inhibiting NLRP3 inflammasome activation.^52^, ^53^ Therefore, the reduction of ROS release might be an effective method to block inflammatory response and treat OA.

Chronic pain is the main symptom of OA, while efficacious treatment of OA pain has not yet been achieved.^54^ The contribution of A3AR in pain states has been evaluated across different studies. A3AR agonists are emerging as promising candidates for neuropathic pain recently.^55^ Activation of A3AR played a key role in the anti‐nociceptive effects at spinal and supraspinal sites.^56^ Additionally, A3AR agonist has been demonstrated to significantly reduce visceral pain,^57^ and prevent the development of paclitaxel‐induced neuropathic pain.^58^ The animal model used in our study is a widely accepted model of surgically induced OA pain.^59^, ^60^ In our present study, oral administration of CF101 was able to relieve secondary mechanical allodynia in an ACLT‐induced rat model of OA. To the best of our knowledge, this is the first study that determines whether CF101 can attenuate nociceptive behaviour in a post‐traumatic OA rat model. The effect of pain amelioration is likely due to the reduced COX‐2 levels in our study. Accordingly, COX‐2 is demonstrated to be involved in inflammation,^61^ and COX‐2 inhibitors are a subclass of NSAIDs that were designed to selectively suppress the production of inflammatory mediators with avoidance gastrointestinal side effects.^62^ In addition, In vitro and in vivo studies have demonstrated the activation and upregulation of NLRP3 in painful conditions including gout and rheumatoid arthritis; however, inhibition of NLRP3 expression can mediate analgesia.^63^ Previous studies have proposed the protective and beneficial effects of A3AR agonists in chronic neuropathic pain via inhibition of NLRP3 associated pathway. A3AR activation has been demonstrated to attenuate neuroinflammation by inhibition of NLRP3 inflammasome activation in a model of traumatic brain injury (TBI),^64^ and A3AR agonists may be useful adjuncts to opioids to manage their unwanted effects, which was associated with reduced dorsal horn of the spinal cord expression of NLRP3 and caspase‐1.^65^ These evidence supports our results that CF101 could relieve OA pain through inhibiting pyroptotic cell death by blocking NLRP3 inflammasome.

Our present study suggests that adenosine A3 receptor activation exerts a chondroprotective effect by inhibiting the NLRP3 inflammasome‐mediated pyroptosis pathway, which not only enriches the mechanism of action of adenosine A3 receptors, but also provides a useful target for the treatment of OA, and offers a potential therapy for OA pain relief. However, the current study has several limitations. First, our study suggests that CF101 could be used to delay ACLT‐induced OA development in a rat model. However, one critical question that we were not able to address is whether CF101 is able to delay OA progression when OA is fully developed. A long‐term study needs to be carried out to investigate it. Second, in order to further determine the safety effect of CF101 on OA, large animals and large animal numbers are needed. Third, we have chosen male animals in the present study. In the future, effects of CF101 in female animals should be assessed due to the hormones difference.

## 5 CONCLUSIONS

Taken together, the findings of our study demonstrate activation of A3AR by CF101 treatment attenuates OA pain and progression of disease in a post‐traumatic animal model. Furthermore, activation of A3AR alleviates inflammatory process through inhibiting ROS initiated NLRP3 inflammasome activation. Therefore, it is reasonable to speculate that activation of A3AR by CF101 and inhibition of NLRP3 inflammasome activation might be a potential therapeutic strategy to prevent OA.

## AUTHOR CONTRIBUTIONS

**Hui Bai:** Conceptualization (lead); data curation (lead); formal analysis (lead); investigation (lead); project administration (supporting); writing – original draft (lead); writing – review and editing (lead). **Zhiheng Zhang:** Conceptualization (lead); data curation (supporting); methodology (supporting); software (supporting); supervision (lead). **Lin Liu:** Investigation (supporting); project administration (supporting); validation (supporting). **Xinyu Wang:** Investigation (supporting); project administration (supporting); writing – review and editing (supporting). **Xiaopeng Song:** Methodology (supporting); validation (supporting); writing – review and editing (supporting). **Li Gao:** Conceptualization (lead); supervision (lead); validation (lead).

## CONFLICT OF INTEREST

The authors declared no conflict of interest.

## Supporting information


Figure S1

## Data Availability

All data generated or analyzed during this study are included in the published article and its supplementary information files.
