# Supplementary figures and images for: Activation of adenosine A3 receptor attenuates progression of osteoarthritis through inhibiting the NLRP3/caspase‐1/GSDMD induced signalling
Source: J Cell Mol Med. 2022 Jun 30;26(15):4230–43. doi: 10.1111/jcmm.17438 (PMC9344816; doi:10.1111/jcmm.17438)

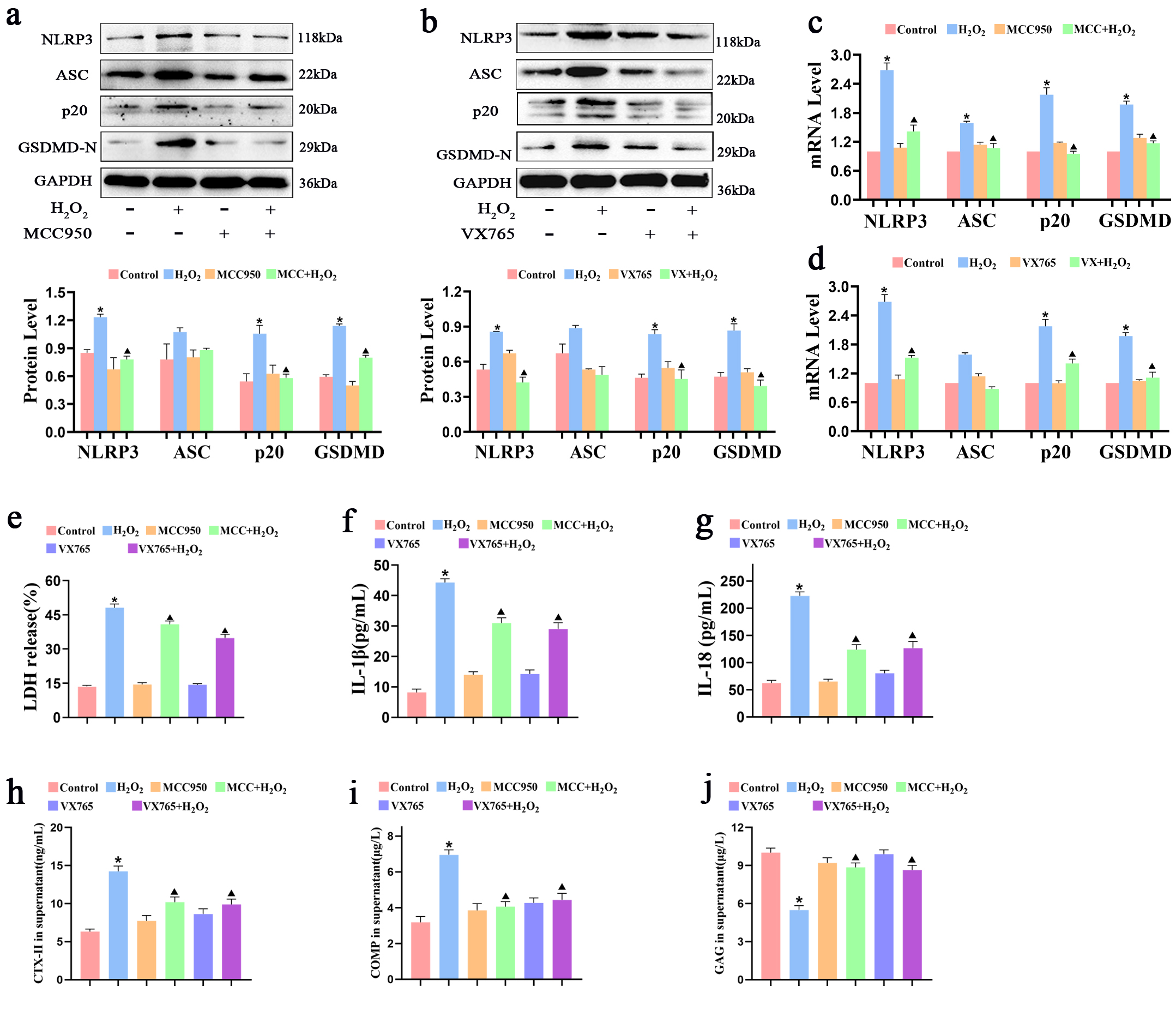

Supplement: Supplementary file 1 — Figure S1 [file JCMM-26-4230-s001.jpg]
